# Learning by stimulation avoidance: A principle to control spiking neural networks dynamics

**DOI:** 10.1371/journal.pone.0170388

**Published:** 2017-02-03

**Authors:** Lana Sinapayen, Atsushi Masumori, Takashi Ikegami

**Affiliations:** The University of Tokyo, Ikegami Laboratory, Tokyo, Japan; Georgia State University, UNITED STATES

## Abstract

Learning based on networks of real neurons, and learning based on biologically inspired models of neural networks, have yet to find general learning rules leading to widespread applications. In this paper, we argue for the existence of a principle allowing to steer the dynamics of a biologically inspired neural network. Using carefully timed external stimulation, the network can be driven towards a desired dynamical state. We term this principle “Learning by Stimulation Avoidance” (LSA). We demonstrate through simulation that the minimal sufficient conditions leading to LSA in artificial networks are also sufficient to reproduce learning results similar to those obtained in biological neurons by Shahaf and Marom, and in addition explains synaptic pruning. We examined the underlying mechanism by simulating a small network of 3 neurons, then scaled it up to a hundred neurons. We show that LSA has a higher explanatory power than existing hypotheses about the response of biological neural networks to external simulation, and can be used as a learning rule for an embodied application: learning of wall avoidance by a simulated robot. In other works, reinforcement learning with spiking networks can be obtained through global reward signals akin simulating the dopamine system; we believe that this is the first project demonstrating sensory-motor learning with random spiking networks through Hebbian learning relying on environmental conditions without a separate reward system.

## Introduction

In two papers published in 2001 and 2002, Shahaf and Marom conduct experiments with a training method that drives rats’ cortical neurons cultivated in vitro to learn given tasks [1, 2]. They show that stimulating the network with a focal current and removing that stimulation when a desired behaviour is executed is sufficient to strengthen said behaviour. By the end of the training, the behaviour is obtained reliably and quickly in response to the stimulation. More specifically, networks learn to increase the firing rate of a group of neurons (output neurons) inside a time window of 50 ms, in response to an external electric stimulation applied to another part of the network (input neurons). This result is powerful, first due to its generality: the network is initially random, the input and output zones’ size and position are chosen by the experimenter, as well as the output’s time window and the desired output pattern. A second attractive feature of the experiment is the simplicity of the training method. To obtain learning in the network, Shahaf and Marom repeat the following two steps: (1) Apply a focal electrical stimulation to the network. (2) When the desired behavior appears, remove the stimulation.

At first the desired output seldom appears in the required time window, but after several training cycles (repeating steps (1) and (2)), the output is reliably obtained. Marom explains these results by invoking the Stimulus Regulation Principle (SRP, from [3, 4]). At the level of neural network, the SRP postulates that stimulation drives the network to “try out” different topologies by modifying neuronal connections (“modifiability”), and that removing the stimulus simply freezes the network in its last configuration (“stability”). The SRP explicitly postulates that no strengthening of neural connections occurs as a result of stimulus removal.

The generality of the results obtained by Shahaf and Marom suggests that this form of learning must be a crucial and very basic property of biological neural networks. But the SRP does not entirely explain the experimental results. Why are several training cycles necessary if “stability” guarantees that the configuration of the network is preserved after stopping the stimulation? How does “modifiability” not conflict with the idea of learning, if we cannot prevent the “good” topology to be modified by the stimulation at each new training cycle?

Importantly, there is no global reward signal sent to the network in Shahaf’s experiment or in our experiments reproducing Shahaf’s results. This is a difference in the object of study between existing papers about learning in spiking networks coupled with a dopamine-like system [5–7] and the present paper. Accepting Shahaf and Marom’s macro phenomenological description of the behavior, we provide a possible mechanism of the behavior at the micro scale: the principle of Learning By Stimulation Avoidance (LSA, [8, 9]). LSA is an emergent property of spiking networks coupled to Hebbian rules [10] and external stimulation. LSA states that the network learns to avoid external stimulus by learning available behaviors e.g. moving away from or destroying the stimulation sources only as a result of local neural plasticity.

In opposition to the SRP, LSA does not postulate that stimulus intensity is the major drive for changes in the network, but rather that the timing of the stimulation relative to network activity is crucial. LSA relies entirely on time dependent strengthening and weakening of neural connections. In addition, LSA proposes an explanatory mechanism for synaptic pruning, which is not covered by the SRP.

LSA emerges from Spike-Timing Dependent Plasticity (STDP), which has been found in both in vivo and in vitro networks. We take STDP as a one basic mechanism governing the neural plasticity [11] and a Hebbian learning rule as a classical realization of STDP in our model. STDP relies on processes so fundamental that it has been consistently found in the brains of a wide range of species, from insects to humans [12–14]. STDP causes changes in the synaptic weight between two firing neurons depending on the timing of their activity: if the presynaptic neuron fires within 20 ms before the postsynaptic neuron, the synaptic weight increases; if the presynaptic neuron fires within 20 ms after the postsynaptic neuron, the synaptic weight decreases.

Shahaf postulates that the SRP might not be at work in “real brains”. Indeed, SRP has not yet been found to take place in the brain, unlike STDP. Although STDP occurs at neuronal level, it has very direct consequences on the sensory-motor coupling of animals with the environment. In vitro and in vivo experiments based on STDP can reliably enhance sensory coupling [15], decrease it [16], and these bidirectional changes can even be combined to create receptive fields in sensory neurons [17, 18].

Therefore, although STDP is a rule that operates at the scale of one neuron, LSA can be expected to emerge at network level in real brains as well as it emerges in artificial networks. LSA at a network level requires an additional condition that is burst suppression. In this paper, we have tested two mechanisms. One is that we add white noise to all neurons and we reduce the number of connections in the network; the other is that we use a Short Term Plasticity rule (STP [19]) that prevents global bursting.

The structure of the paper is as follows: we show that the conditions necessary to obtain LSA are sufficient to reproduce biological results, study the dynamics of LSA in a minimal network of 3 neurons and present burst suppression methods in Section 1. We show that LSA works in a scaled up network of 100 neurons with burst suppression by additive noise in Section 2. We show that LSA also works with burst suppression by STP with 100 neurons in Section 3, even when there are no direct connections between input and output neurons. Finally we implement a simple embodied application using LSA and STP for burst suppression in a simulated robot in Section 4.

## 1 LSA is sufficient to explain biological results

In [8] we showed that a simulated random spiking network built from [20, 21] combined to STDP could be driven to learn desired output patterns using a training method similar to that of Shahaf et al. Shahaf shows that his training protocol can reduce the response time of a network. The response time is defined as the delay between the application of the stimulation and the observation of a desired output from the network. In Shahaf’s first series of experiments (“simple learning” experiments), the desired output is defined by the fulfillment of one condition:

Condition 1: the electrical activity must increase in a chosen Output Zone A. This is the experiment we reproduced in [8], demonstrating that this learning behaviour is a direct effect of STDP and is captured by the principle of LSA: firing patterns leading to the removal of external stimulation are strengthened, firing patterns that lead to the application of an external stimulation are avoided.

In this section we show that the same methods are sufficient to obtain results similar to the second series of experiments performed by Shahaf (“selective learning” experiments), in which the desired output is the simultaneous fulfillment of Condition 1 as defined before and a second condition:

Condition 2: a different output zone (Output Zone B) must not exhibit enhanced electrical activity. When both conditions are fulfilled, the result is called selective learning because only Output Zone A must learn to increase its activity inside the time window, while Output Zone B must not increase its activity. We reproduce the experiment as follows.

### 1.1 Network model

**Fig 1:**
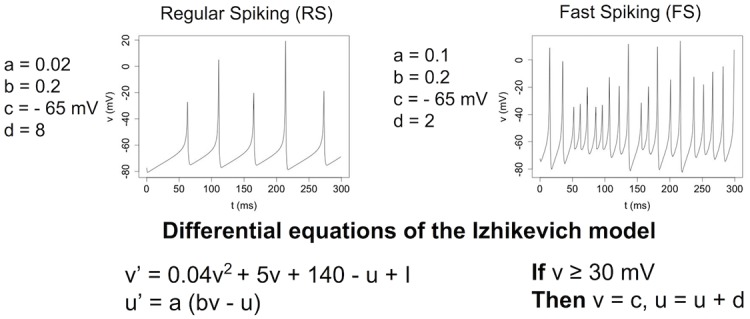
Equations and dynamics of regular spiking and fast spiking neurons simulated with the Izhikevich model. Equations and dynamics of regular spiking and fast spiking neurons simulated with the Izhikevich model.

We use the model of spiking neuron devised by Izhikevich [22] to simulate excitatory neurons (regular spiking neurons) and inhibitory neurons (fast spiking neurons) with a simulation time step of 1 ms. The equations of the neural model and the resulting dynamics are shown in Fig 1. We simulate a fully connected network of 100 neurons (self-connections are forbidden) with 80 excitatory and 20 inhibitory neurons. This ratio of 20% of inhibitory neurons is standard in simulations [20, 22] and close to real biological values (15%, [23]). The initial weights are random (uniform distribution: 0 < *w* < 5 for excitatory neurons, −5 < *w* < 0 for inhibitory neurons). LSA may have different features with different network topologies and time delays; however, we believe that the conditions simulated here are the simplest setup for having LSA. The neurons receive three kinds of input: (1) Zero-mean Gaussian noise *m* with a standard deviation *σ* = 3 mV is injected in each neuron at each time step; (2) External stimulation *e* with a value of 1 mV and a frequency of 1000 Hz. The external stimulation is stopped when the network exhibits the desired output. (3) Stimulation from other neurons: when a neuron *a* spikes, the value of the weight *w*_*a*,*b*_ is added as an input for neuron *b* without delay. All these inputs are added for each neuron *n*_*i*_ at each iteration as:

$$\begin{array}{c} I_{i}=I_{i}^{*}+e_{i}+m_{i}\;. \end{array}$$ (1)

$$\begin{array}{ccc} I_{i}^{*} & = & \sum_{j=0}^{n}w_{j,i}\times f_{j}\;,\\ f_{j} & = & \left\{ \begin{array}{cc} 1, & \text{if}\;\text{neuron}\;\text{j}\;\text{is}\;\text{firing}\\ 0, & \text{otherwise}. \end{array}\right. \end{array}$$ (2)

**Fig 2:**
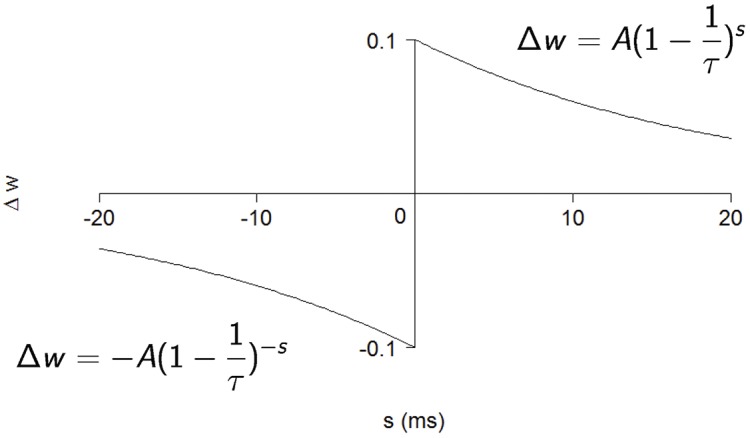
The Spike-Timing Dependent Plasticity (STDP) function governing the weight variation Δ*w* of the synapse from neuron *a* to neuron *b* depending on the relative spike timing *s* = *t*_*b*_ − *t*_*a*_. *A* = 0.1; *τ* = 20 ms.

We add synaptic plasticity in the form of STDP as proposed in [19]. STDP is applied only between excitatory neurons; other connections keep their initial weight during all the simulation. We use additive STDP: Fig 2 shows the variation of weight Δ*w* for a synapse between connected neurons. As shown on the figure, Δ*w* is negative if the post-synaptic neuron fires first, and positive the pre-synaptic neuron fires first. The total weight *w* varies as:

$$\begin{array}{c} w_{t}=w_{t-1}+\Delta w\;. \end{array}$$ (3)

The maximum possible value of weight is fixed to *w*_*max*_ = 10. if *w* > *w*_*max*_, *w* is reset to *w*_*max*_. In the experiments with 100-neurons networks, we also apply a decay function to all the weights in the network. The decay function is applied at each iteration *t* as:

$$\begin{array}{c} \forall w_{t},\;w_{t+1}=\left(1-\mu \right)w_{t} \end{array}$$ (4)

We fix the decay parameter as *μ* = 5 × 10^−7^.

### 1.2 Superficial selective learning experiment

In this section we reproduce in simulation the biological results obtained by Shahaf. A group of 10 excitatory neurons are stimulated. Two different groups of 10 neurons are monitored (Output Zone A and Output Zone B). We define the desired output pattern as: *n* > = 4 neurons spike in Output Zone A (Condition 1), and *n* < 4 neurons spike in Output Zone B (Condition 2). Both conditions must be fulfilled simultaneously, i.e. at the same millisecond. We stop the external stimulation as soon as the desired output is observed. If the desired output is not observed after 10,000 ms of stimulation, the stimulation is also stopped. After a random delay of 1,000 to 2,000 ms, the stimulation starts again.

There are important differences with the biological experiment: the stimulation frequency (Shahaf uses lower frequencies), its intensity (this parameter is unknown in Shahaf’s experiment) and the time window for the output (in Shahaf’s results the activity of Output Zone A is arguably higher even outside of the selected output window). We also use a fully connected network, while the biological network grown in vitro is likely to be sparsely connected [24].

**Fig 3:**
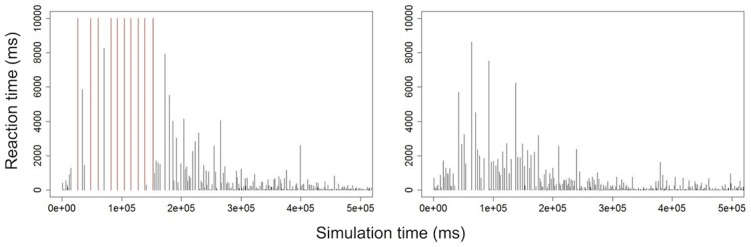
Evolution of the reaction times of 2 successful neural networks at the selective learning task. 20 networks were simulated, 18 of which successfully learned the task (Table 1). Red lines represent when there was no response from the network. A learning curve is clearly visible.

Despite these differences, we obtain results comparable to those of Shahaf: the reaction time, initially random, becomes shorter with training (Fig 3). We also perform the experiment with no stimulation at all and find a success rate of 0%; the statistics of the selective learning experiment are summarized in Table 1.

**Table 1:** Statistical performance of the network.

Condition	Success rate	Learning time	Attained reaction time
Selective learning	90%	187 ± 16 s	389 ± 54 ms
No external stim.	0%	–	–

Learning time: the task is learned when the reaction time of the network reaches a value inferior to 4,000 ms and keeps under this limit. The success rate is the percentage of networks that successfully learned the task in 400,000 ms or less (N = 20 networks per condition). The attained reaction time is calculated for successful networks after learning. Standard error is indicated.

As shown by these results, the network exhibits selective learning as defined by Shahaf. But we also find that despite a success rate of 90% at exhibiting the desired firing pattern, both the firing rates of Output Zone A and Output Zone B increase in equivalent proportions: the two output zones fire at the same rate but in a desynchronized way. The task was to activate Output Zone A and suppress the activity in Output Zone B. But the opposite result also occurs at the same time, in an opposite phase. Although data about firing rates is not specifically discussed in Shahaf’s paper, it is possible that bursting did happen. Shahaf himself reports in his experiment that only half of the in-vitro networks succeeded at selective learning, while all succeeded at the “simple learning” task. Our hypothesis is that bursts are detrimental to learning [25] and explain the difficulty of obtaining selective learning. If this hypothesis is true, burst suppression is essential to obtain learning. We explain why burst suppression is necessary by first explaining how learning works in a small network. Then we study 100-neurons networks with global bursting suppression.

### 1.3 Dynamics of LSA in a minimal network

In [8] we showed that a minimal network of 2 neurons consistently follows the principle of LSA; we also showed that a single neuron is able to prune one synapse and enhance another synapse simultaneously depending on the stimulation received by the two presynaptic neurons. In this experiment we examine the weights dynamics in a chain of 3 excitatory neurons all connected to each other: one neuron is used as input, one as output, and they are separated by a “hidden neuron”.

**Fig 4:**
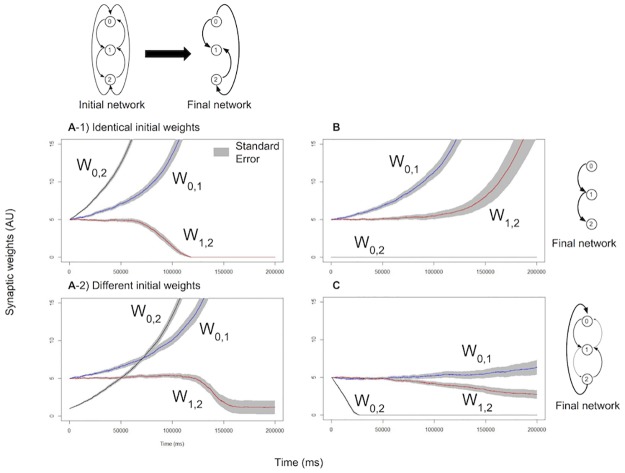
Dynamics of weight changes induced by LSA in small networks of 3 neurons. A) Reinforcement: spiking of neuron 2 stops the stimulation in neuron 0. The direct weight *w*_0,2_ grows faster than other weights, even when starting at a lower value. B) Artificially fixing the direct weight *w*_0,2_ to 0, a longer pathway of 2 connections 0 → 1 → 2 is established. C) Pruning: spiking of neuron 2 starts external stimulation to neuron 0. As a result, *w*_0,2_ is pruned.

Neurons are labeled 0 (input neuron), 1 (hidden neuron) and 2 (output neuron). Fig 4 shows the results of experiments with different learning conditions and different initial states. The results can be summarized as follows: (1) In the reinforcement condition, direct connections between input and output are privileged over indirect connections. All connections are updated with the same time step (1 ms), therefore the fastest path (direct connection) will always cause neuron 2 to fire before the longer path (made of several connections) can be completely activated. When no direct connection exists, weights on longer paths are correctly increased. (2) LSA explains a behaviour that is not discussed in the SRP: synapse pruning. LSA predicts that networks evolve as much as possible towards dynamical states that cause the less external stimulation. Here LSA only prunes weights of direct connections between the input and output, as this is sufficient to stop all stimulation to the output neuron. (3) For neurons that are strongly stimulated (here, neuron 0) the default behaviour of the output weights is to increase, except if submitted to the pruning influence of LSA. Neurons that fire constantly bias other neurons to fire after them, automatically increasing their output weights.

This raises concerns about the stability of larger, fully connected networks; all weights could simply increase to the maximum value. But introducing inhibitory neurons in the network can improve network stability [26]. In our experiments with 100-neuron networks, 20 are inhibitory neurons with fixed input weights and output weights. In addition, we make the hypothesis that global bursts in the network can impair LSA, as all neurons fire together make it impossible to tease apart individual neuron’s contributions to the postsynaptic neuron’s excitation. Global bursts are also considered to be a pathological behaviour for in vitro networks, and do not occur with healthy in vivo networks [25].

In the remainder of this paper, we use two different methods to obtain burst suppression. The first method is to add strong noise to the neurons and to reduce the initial number of connections in the network. This produces a desynchronization of the network activity. In Section 2, we show that this method allows LSA to work in networks of 100 neurons. The second method is to apply Short Term Plasticity to all the connections in the network. In Section 3 and 4 we show that this method of burst suppression allows proper selective learning even in the absence of direct connections between input and output; we also show an application to a robot experiment.

## 2 Burst suppression by adding noise

### 2.1 Selective learning with burst suppression

**Fig 5:**
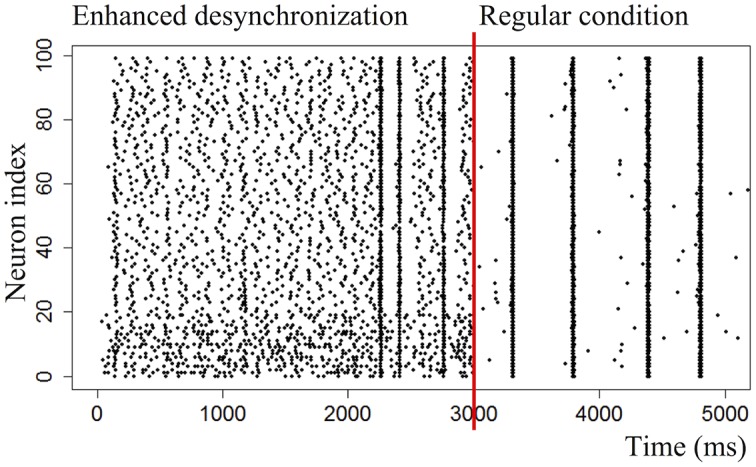
Raster plots of a regular network (activity concentrated in bursts) and a network of which parameters have been tuned to reduce bursting and enhance desynchronized spiking. The desynchronizing effect of sparsely connecting the network and increasing the noise are clearly visible.

In this experiment, burst suppression is obtained in the 100-neuron network by reducing the number of connections: each neuron has 20 random connections to other neurons (uniform distribution, 0 < *w* < 10), a high maximum weight of 50, high external input *e* = 10 mV and high noise *σ* = 5 mV. Variations in the number of connections and the weight variance are examined later in this paper. These networks are less prone to global bursts and exhibit strong desynchronized activity, as shown in Fig 5.

We monitor two output zones and fix two independent stimulation conditions:

(Stop Condition) Input Zone A is stimulated. After *n* > = 1 neurons in Output Zone A spike, the external stimulation to Input Zone A is stopped. If the desired output is not observed after 10,000 ms of stimulation, the stimulation is also stopped. After a random delay of 1,000 to 2,000 ms, the stimulation starts again.

(Stimulus Condition) After *n* > = 1 neurons spike in Output Zone B, the whole network (excluding inhibitory neurons and Output Zone B itself) is stimulated for 10 ms. The goal is to obtain true selective learning, by increasing the weights to Output Zone A and prune those to Output Zone B, therefore obtaining different firing rates. This Stimulus Condition is opposite to the Stop Condition. It requires stimulus when a neuron in the output region fires. Here we use the minimal threshold (= 1) for the Stop Condition, but for later experiments we use a threshold of 4 neurons.

Only a few (comparatively to the network size) spiking presynaptic neurons are necessary to make a postsynaptic neuron fire if the connection weights are high. In consequence, the Stimulus Condition must be able to prune as many input synapses to Output Zone B as possible. It is therefore important to suppress global bursts: they cause Output Zone B to fire at the same time as the whole network, making it impossible to update only relevant weights without also updating unrelated weights.

**Fig 6:**
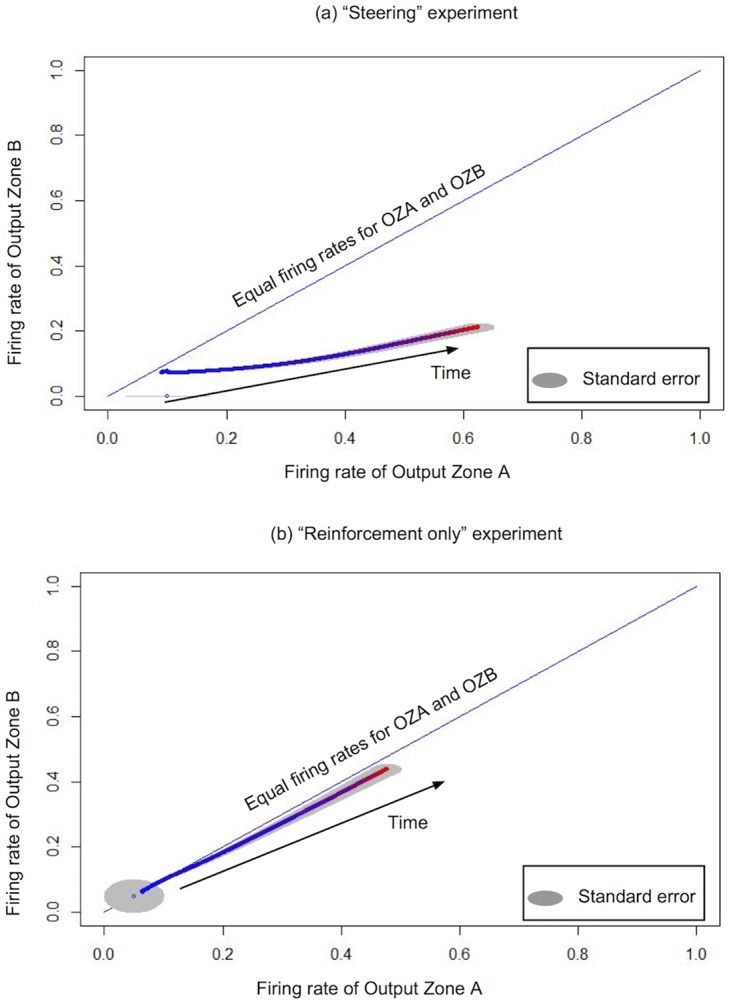
Trajectory of the network in the two-dimensional space of the firing rates of Output Zone A and Output Zone B. Using LSA, we can steer the network in this space; by contrast, the “reinforcement only” experiment maintains the network balanced relatively to the two firing rates. (a) leads to the low external stimulation region but (b) does not. Statistical results for N = 20 networks.

As a result of LSA, the network must move from a state where both output zones fire at the same rate, to a state where Output Zone B fires at lower rates and Output Zone A fires at higher rates. This prediction is realized, as we can see in Fig 6a: the trajectory of firing rates goes to the space of low external stimulation. In Fig 6b, we show for comparison the trajectory for networks with only the Stop Condition applied: on average the firing rates of both output zones are equivalent, with individual networks trajectories ending up indiscriminately at the top left or bottom right of the space.

These results could potentially be reproduced in a network in vitro: the Izhikevich model of spiking network that we use has been found to exhibit the same dynamics as real neurons, and our experiments with STDP can reproduce some results of biological experiments; therefore there is a probability that this results predicted by LSA still holds in biological networks with suppressed bursts, especially since we have shown that LSA gives promising results on biological networks embodied in simple robots [9].

### 2.2 Parameter exploration

We perform a parameter search to explore the working conditions of the “simple learning” task. In this section, we vary the number of connections in the network and the variance *v* of the weights. For each neuron an output connection is chosen at random and the weight is initialised at *w* = 5 + *ω* (*w* = −5 + *ω*), with *ω* following a uniform distribution between −*v* and *v*. This process is repeated *M* times for each neuron, 0 < *M* < 150. The same connection can be chosen twice at random, so the actual number of connections can be inferior to *M*.

**Fig 7:**
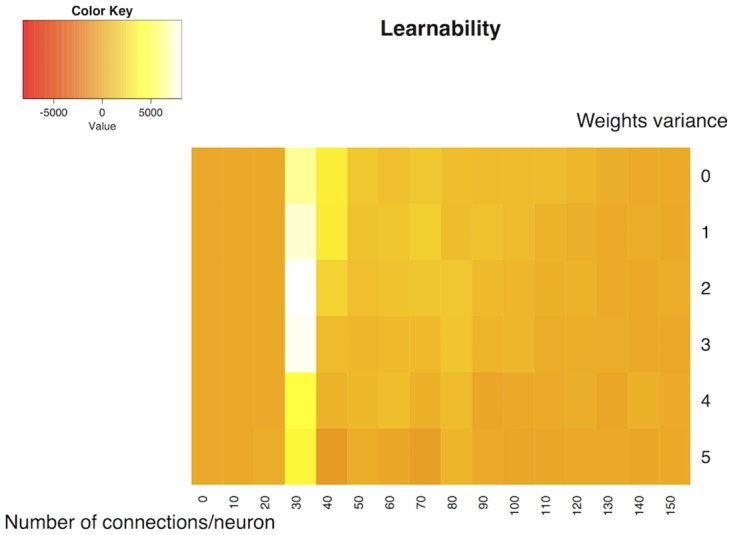
Performance of learning depending of network connectivity and initial weights variance. Learnability is defined as the average difference between the firing rate of the Output Zone during the first 100 seconds and the last 100 seconds. The ideal region of the parameter space to obtain good learning results is between 20 and 30 connections per neuron. By comparison, the variance has less influence. Statistical results for N = 20 networks for each parameter set.

For each set (*ω*, *M*) we perform N = 20 experiments (with Stop Condition but no Stimulus Condition) of length T = 500 seconds. Learnability is defined as the average difference between the firing rate of the Output Zone during the first 100 seconds and the last 100 seconds and is reported on the heat map Fig 7. This figure shows that the variance in the initial weights has low influence on the final learning results, but the ideal region to obtain good learning results is between 20 and 30 connections per neuron. Above these values, the increased connectivity of the network might cause too many bursts, affecting the learning results. Below these values, we there may not be a path connecting the input neurons to the output neurons, making the learning task impossible. This does not mean that direct connections between input and output are necessary to obtain learning: in the next section we show that LSA works even without direct connections between input and output.

## 3 Burst suppression by short term plasticity

In this section we set all weights from input neurons to output neurons to 0. The initial weights are random (uniform distribution: 0 < *w* < 5 for excitatory neurons, −5 < *w* < 0 for inhibitory neurons). Zero-mean Gaussian noise *m* with a standard deviation *σ* = 3 mV is injected in each neuron at each time step. The maximum possible value of weight is fixed to *w*_*max*_ = 20. The external stimulation value is *e* = 10 mV. Burst suppression is obtained by adding a phenomenological model of Short Term Plasticity (STP, [27]) to the network, as a way to suppress bursts despite the network being fully connected. STP is a reversible plasticity rule that decreases the intensity of neuronal spikes if they are too close in time, preventing the network to enter a state of global synchronized activity (details in the Appendix).

As in Section 1.2 the goal is for Output Zone A to increase its firing rate and Output Zone B to decrease it. The conditions are as follows:

(Stop Condition) Input Zone A is stimulated. After *n* > = 4 neurons spike in Output Zone A and if only *n* < 4 neurons spiked in Output Zone B, the external stimulation to Input Zone A is stopped. After a random delay of 1,000 to 2,000 ms, the stimulation starts again.

(Stimulus Condition) After *n* > = 1 neurons in Output Zone B spike, the whole network (excluding inhibitory neurons and Output Zone B itself) is stimulated for 10ms.

**Fig 8:**
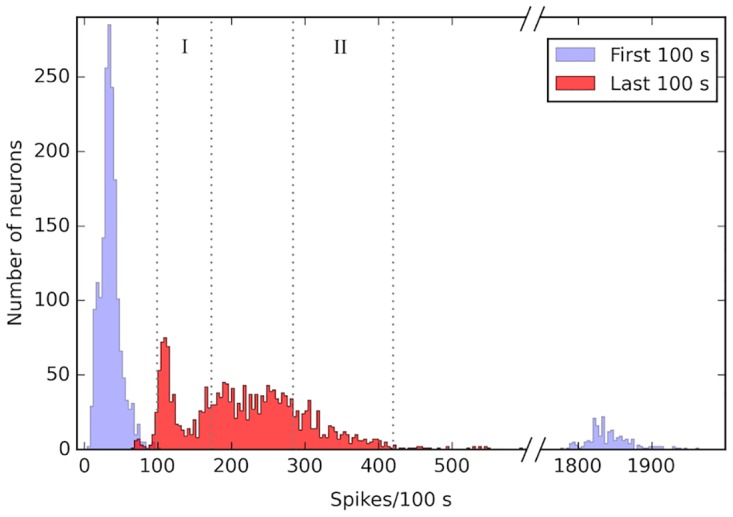
Firing rate distribution. This figure shows the cumulative number of neurons in each firing rate bin for 20 networks. Before learning (first 100 s, blue), most neurons have a very low firing rate (leftmost peak) except for the input neurons (rightmost peak). After learning (last 100 s, red), the neurons are distributed in 2 groups: low firing rate (2nd peak from the left) and medium firing rate. 50% of the Output Zone B neurons are contained in the zone marked I; 50% of the Output Zone A neurons are contained in the zone marked II.

The conditions are therefore stricter than in Section 2.1. Fig 8 shows the distribution of firing rates before and after learning the task. Before learning, only the input neurons have a high firing rate due to the external simulation. After learning, 50% of the Output Zone A is contained in the highest firing rate zone (region II of Fig 8), and 50% of the Output Zone B in the lowest firing rate zone (region I). Even if the stimulus condition is controlled by a single neuron in the region B, the average firing rate in output regions A and B is sufficiently distinguished as is shown in Fig 8.

This experiment shows that LSA works with a different burst suppression method, even when direct input-output connections are cut. In the next section, we propose a simple application of LSA in a simulated robot.

## 4 Embodied application: wall avoidance with a robot

We show that LSA can be used in a practical embodied application: wall avoidance learning. The principle of this experiment is that a robot has distance sensors that stimulate the network when the robot is close to walls: the more the robot learns to avoid walls, the less stimulation it receives. Burst suppression is achieved through STP.

**Fig 9:**
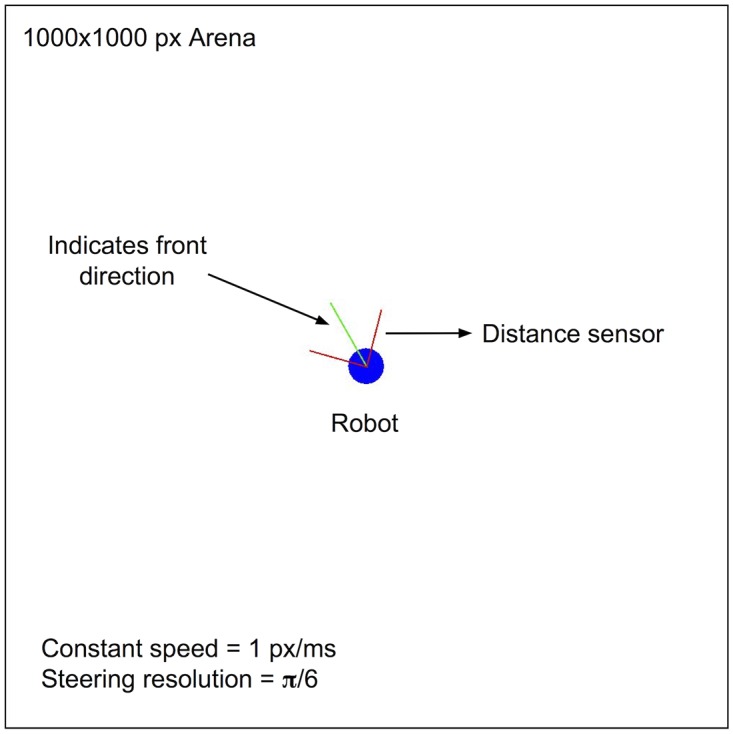
Robot simulation. The robot has distance sensors and must learn to stay away from the arena’s walls.

We simulate a simple robot moving inside a closed arena. The robot has two distance sensors on the front (right and left), allowing it to detect walls (Fig 9). A 100-neuron network takes the two sensors’ values as respective input for two input zones (10 excitatory neurons each). Activity in two output zones of the network (10 excitatory neurons each) allows the robot to turn right or left. In these conditions, steering when encountering a wall can stop the stimulation received by the input zones from the distance sensors, if the new direction of the robot points away from the walls. The behaviour enhanced by LSA should therefore be wall avoidance. We call this experiment a “closed loop” experiment, because steering away from the walls automatically stops the external stimulation at the right timing.

The arena is a square of size 1000 px (pixels). The robot is a 25 px radius circle, constantly moving at 1 px/ms except in case of collision with a wall. The robot has two distance sensors oriented respectively at *π*/4 and −*π*/4 from the front direction of the robot. The sensors have a range of 80 px; they are activated when the robot is at less than 80 px from a wall, on the direction supported by each sensor’s orientation. Two input zones in the network (10 neurons each) receive input in mV at a frequency of 1000 Hz from the sensors as *input* = *sensitivity*/*distance*.

The *sensitivity* of the sensors is fixed at a constant value for the duration of each experiment. For simplicity, the robot’s steering is non-differential. It is controlled by the spikes of two output zones in the network (10 neurons each). For each spike in the left output zone, the robots steers *π*/6 radian (to the left); for each spike in the right output zone, the robots steers −*π*/6 radian (to the right).

**Fig 10:**
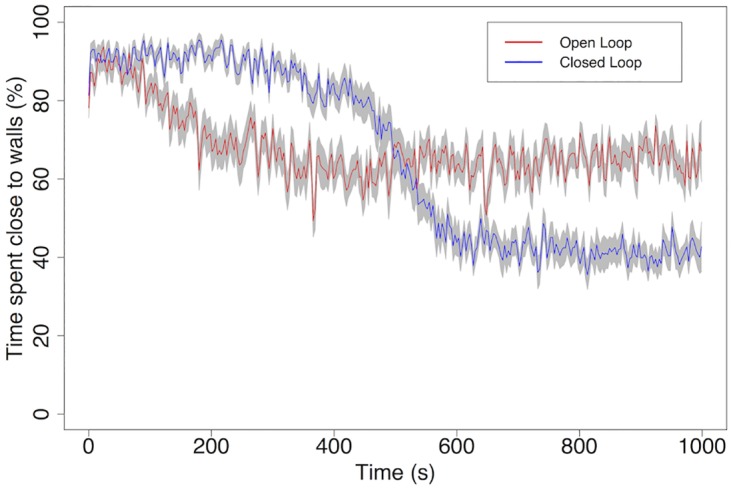
Learning curves of the wall avoidance task. The robot is considered to be “close” to a wall if it is less than 80 pixels from the wall, which corresponds to the range of its distance sensors. The results show that random steering due to high random activity in the network leads to spending 64% of the time close to walls, while learning due to LSA leads to only 43% time spent close to walls. Statistical results for N = 20 networks, standard error is indicated.

We compare the results of this experiment (closed loop experiment, sensor *sensitivity* = 8 mV) with a control experiment where a constant stimulation (8 mV) is applied to the network’s input zones, independently of the distance or orientation of the robot relative to the walls (open loop experiment). Both conditions are tested 20 times and averaged. Fig 10 shows two important effects: (1) Constant stimulation leads to higher activity in the network, provoking random steering of the robot which leads to some level of wall avoidance, but (2) Closed loop feedback is necessary to obtain actual wall avoidance learning. Indeed, by the end of the closed loop experiment the robot spends only 43% of its time at less than 80 pixels from any wall (the range of the distance sensors), against 64% in the open loop experiment. The importance of feedback over simply having high stimulation is further demonstrated by the fact that the open loop robot is receiving overall a greater amount of stimulation than the closed loop robot. At 400 s, when the learning curve of the closed loop robot starts sloping down, the robot has received on average 1.54 mV of stimulation per millisecond. The open loop robot, which by that time has reached its best performance, has received 16 mV/ms. In a different experiment, we give to open loop robots the same amount of average stimulation that is received by closed loop robots; by the end of the experiment (1000 s) the open loop robots still spend more than 80% of the time close to arena walls.

**Fig 11:**
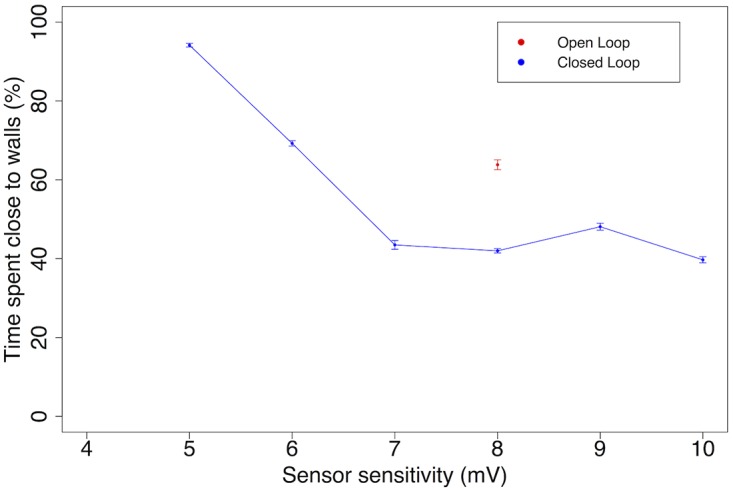
Learnability of wall avoidance based on sensor sensitivity. High numbers on the *x* axis indicate high sensitivity. Under 5 mV, the task cannot be learned; learnability improves until 7 mV, leading to the maximum performance of the robot. The open loop and closed loop results for the same sensitivity of 8 mV are reported from Fig 10. We have omitted the open loop cases as they are insensitive to the walls, but shown for the case at the sensor sensitivity = 8 mV. Statistical results for N = 20 networks with standard error.

We further study the effect of stimulation strength and feedback on learning performance by varying the sensitivity of the distance sensors. The average state in the last 300 seconds of each task (total duration: 1000 s) is reported on Fig 11. The open loop result of the previous experiment is included for reference. Fig 11 indicates that the learnability of the task is improved by having more sensitive sensors, up to a limit of about 40%. Having sensors with a sensitivity of more than 7 mV does not improve the performance of the robot. This result is in direct contradiction with the SRP’s leading hypothesis, which postulates that the intensity of stimulation is the driving force behind network modification. If that was the case, more sensitive sensors should always lead to better learnability. By contrast, LSA emphasizes timing, not strength of the simulation. The 40% limit could be due in part to unreliable feedback, as steering away from a wall can put the robot directly in contact with another wall if it is stuck in a corner: the same action can lead to start or removal of stimulation depending on the context.

## Discussion

In this paper, we introduce LSA, a new principle explaining the dynamics of spiking neural networks under the influence of external stimulation. LSA is not just an another theoretical neural learning rule, but provides a new interpretation to the learning behavior of neural cells in vitro. In particular LSA is most efficient for sensory-motor coupling systems. The model presented in this paper is very simplified compared to biological neurons, as it uses only two types of neurons, one type of STDP, no homeostatic mechanism, etc. Nevertheless, the model is able to reproduce key features of experiments conducted with biological neurons by Shahaf et al. and explain results obtained in vitro with neurons submitted to external stimulation. LSA also offers an explanation to a biological mechanism that is ignored by the theory of SRP, namely the pruning of synapses. LSA has direct practical applications: by engineering causal relationships between neural dynamics and external stimulation, we can induce learning and change the dynamics of the neurons from the outside.

LSA relies on the mechanism of STDP, and we demonstrated that the conditions to obtain LSA are: (1) Causal coupling between neural network’s behaviour and environmental stimulation; (2) Burst suppression. We obtain burst suppression by increasing the input noise in the model or by using STP. We assume that in healthy biological neurons, the neuronal noise may be introduced by spontaneous neuronal activity. As we have shown, LSA does not support the theory of the Stimulus Regulation Principle. It could be closer to the Principle of Free Energy Minimisation introduced by Friston [28]. The Free Energy Principle states that networks strive to avoid surprising inputs by learning to predict external stimulation. An expected behaviour of networks obeying the Free Energy Principle, or obeying LSA is that they can fall into the dark room paradox, avoiding incoming input by cutting all sources of external simulation. A key difference between LSA and the Free Energy Principle is that our network does not predict incoming input. Most importantly, LSA automatically let stimuli from environment terminate at the right timing, so that a network can self-organize using environmental information.

## Appendix

STP is a reversible plasticity rule that decreases the intensity of neuronal spikes if they are too close in time, preventing the network to enter a state of global synchronized activity. As in the original paper, we apply STP to the output weights from excitatory neurons to both excitatory and inhibitory neurons.

$$\begin{array}{c} w_{i,j}^{*}=uxw_{i,j} \end{array}$$ (5)

$$\begin{array}{c} \frac{dx}{dt}=\frac{1-x}{\tau_{d}}-uxf_{i} \end{array}$$ (6)

$$\begin{array}{c} \frac{du}{dt}=\frac{U-u}{\tau_{f}}+U\left(1-u\right)f_{i} \end{array}$$ (7)

where the initial release probability parameter *U* = 0.2, and *τ*_*d*_ = 200 ms and *τ*_*f*_ = 600 ms are respectively the depression and facilitation time constants. Briefly speaking, *x* is a fast depression variable reducing the amplitude of the spikes of neurons that fire too often, while *u* is a slow facilitation variable that enhances the spikes of these same neurons. As a result of the interplay of *x* and *u*, neurons constantly firing at high frequency are inhibited, while neurons irregularly firing at high or low frequency are unaffected (the maximum value of *ux* is 1). STP acts as a short term reversible factor on the original synaptic weight, with the side effect of preventing global bursting of the network. Eq (2) becomes

$$\begin{array}{ccc} I_{i}^{*} & = & \sum_{j=0}^{n}w_{j,i}^{*}\times f_{j}\;,\\ f_{j} & = & \left\{ \begin{array}{cc} 1, & \text{if}\;\text{neuron}\;\text{j}\;\text{is}\;\text{firing}\\ 0, & \text{otherwise}. \end{array}\right. \end{array}$$ (8)
